# Azathioprine with Allopurinol: Lower Deoxythioguanosine in DNA and Transcriptome Changes Indicate Mechanistic Differences to Azathioprine Alone

**DOI:** 10.1097/MIB.0000000000001131

**Published:** 2017-04-27

**Authors:** Sally A. Coulthard, Phil Berry, Sarah McGarrity, Simon McLaughlin, Azhar Ansari, Christopher P. F. Redfern

**Affiliations:** * Institute of Cellular Medicine, Newcastle University, Newcastle upon Tyne, United Kingdom;; † Northern Institute of Cancer Research, Newcastle University, Newcastle upon Tyne, United Kingdom;; ‡ Department of Gastroenterology, Royal Bournemouth Hospital, Bournemouth, United Kingdom;; § Faculty of Health and Social Sciences, Bournemouth University, Bournemouth, United Kingdom;; ‖ Gastroenterology Department, East Surrey Hospital, Redhill, East Surrey, United Kingdom; and; ¶ School of Bioscience, University of Surrey, Guildford, United Kingdom.

**Keywords:** azathioprine, thiopurine, allopurinol

## Abstract

**Background:**

Use of azathioprine (AZA) for inflammatory bowel disease is limited by side effects or poor efficacy. Combining low-dose azathioprine with allopurinol (LDAA) bypasses side effects, improves efficacy, and may be appropriate as first-line therapy. We test the hypothesis that standard-dose azathioprine (AZA) and LDAA treatments work by similar mechanisms, using incorporation of the metabolite deoxythioguanosine into patient DNA, white-blood cell counts, and transcriptome analysis as biological markers of drug effect.

**Methods:**

DNA was extracted from peripheral whole-blood from patients with IBD treated with AZA or LDAA, and analyzed for DNA-incorporated deoxythioguanosine. Measurement of red-blood cell thiopurine metabolites was part of usual clinical practice, and pre- and on-treatment (12 wk) blood samples were used for transcriptome analysis.

**Results:**

There were no differences in reduction of white-cell counts between the 2 treatment groups, but patients on LDAA had lower DNA-incorporated deoxythioguanosine than those on AZA; for both groups, incorporated deoxythioguanosine was lower in patients on thiopurines for 24 weeks or more (maintenance of remission) compared to patients treated for less than 24 weeks (achievement of remission). Patients on LDAA had higher levels of red-blood cell thioguanine nucleotides than those on AZA, but there was no correlation between these or their methylated metabolites, and incorporated deoxythioguanosine. Transcriptome analysis suggested down-regulation of immune responses consistent with effective immunosuppression in patients receiving LDAA, with evidence for different mechanisms of action between the 2 therapies.

**Conclusions:**

LDAA is biologically effective despite lower deoxythioguanosine incorporation into DNA, and has different mechanisms of action compared to standard-dose azathioprine.

## Abstract 2

Article first published online 27 April 2017

Supplemental Digital Content is Available in the Text.

Azathioprine, a thiopurine prodrug of 6-mercaptopurine (6-MP), is an established long-term, safe, and effective treatment for inflammatory bowel disease (IBD). However, many patients suffer from thiopurine-related side effects, including hepatotoxicity, which the coadministration of low-dose azathioprine with the xanthine oxidase inhibitor allopurinol (LDAA) is an accepted strategy for avoiding.^1,2^ The clinical use of LDAA includes patients who develop abnormal liver function tests or side effects to standard-dose azathioprine or 6-MP. It is also used in a few patients with a poor response to azathioprine/6-MP, and a specific metabolite profile characterized by high methylated metabolite (MMP) and low thioguanine nucleotide (TGNs) levels in red-blood cells (RBC).^3,4^ This “hypermethylation” profile may result from preferential methylation of 6-MP by thiopurine methyltransferase (TPMT) to MMP (Fig. 1), giving a poor clinical response and increased chance of hepatotoxicity.^5–8^ Methylated thiopurine metabolites make an important contribution to thiopurine toxicity^9^; patients with a hypermethylator profile or hepatotoxicity respond to LDAA with an increase in RBC TGNs, a reduction in RBC MMP, and disease remission.^4,10,11^

**FIGURE 1.**
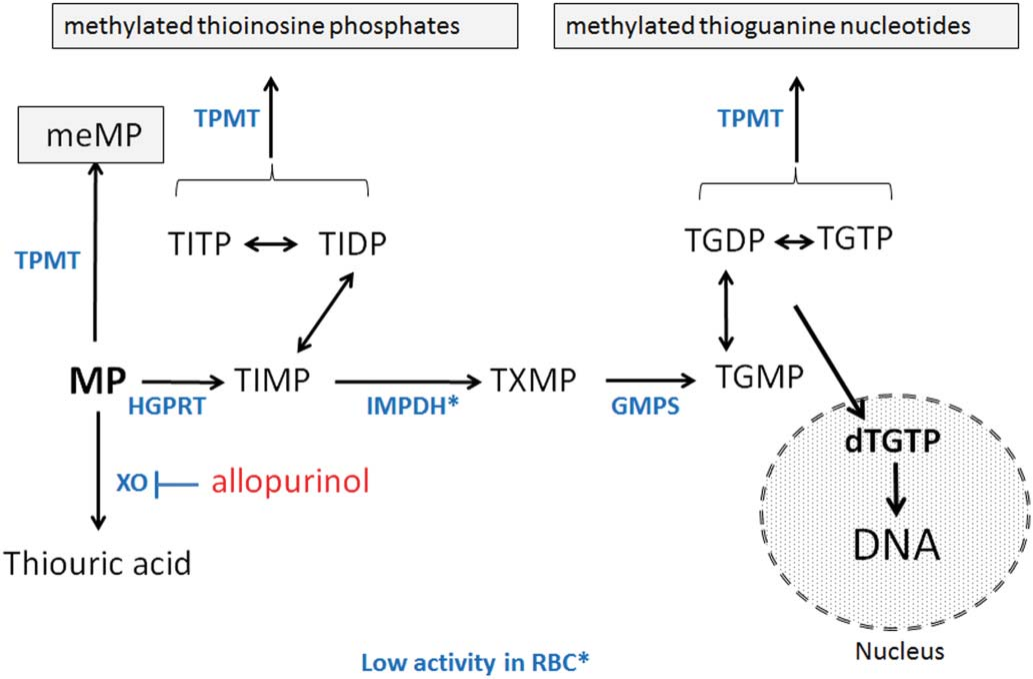
Schematic outline of thiopurine metabolism. Azathioprine is nonenzymatically cleaved to 6-MP which is metabolized by the enzymes (blue text) inosine-monophosphate dehydrogenase (IMPDH), thiopurine methyltransferase (TPMT), hypoxanthine-guanine phosphoribosyl transferase (HGPRT), inosine tri-phosphatase (ITPase) and guanosine monophosphate synthetase (GMPS). MeMP, methylmercaptopurine; TIMP, thioinosine monophosphate with metabolism to the di- and triphosphates TIDP and TITP, respectively; TXMP, thioxanthine monophosphate; TGMP, thioguanosine monophosphate with metabolism to the di- and triphosphates TGDP and TGTP, respectively, and incorporation into DNA. 6-MP is inactivated by metabolism to thioxanthine then thiouric acid via xanthine oxidase (XO), which is inhibited by allopurinol. Thioxanthine may inhibit TPMT. The molecular mechanism of TGN immunosuppression may include G-protein (rac-1) inhibition.

In a recent open-label study, Pavlidis et al^12^ treated a cohort of thiopurine-naive IBD patients with LDAA, dose-adjusted for TPMT status, and without prior selection based on RBC metabolite levels. Clinical response in that study was seen in 45/57 (79%) patients with Crohn's disease and 34/53 (64%) patients with ulcerative colitis/IBD-unclassified, with remission rates of 61% and 53% of patients, respectively. This is further evidence that the use of LDAA both in hypermethylators^3,4,10,11^ and when used as a first-line therapy without metabolic screening^12^ is an effective clinical strategy comparable to established guidelines for IBD.^13^

The aim of this pilot study was to test the hypothesis that the AZA and LDAA treatment strategies are equivalent in terms of biological markers of thiopurine effect. As markers, we have investigated the utility of DNA-incorporated deoxythioguanosine (dTG^DNA^) in comparison with RBC TGN measurement and reduction in neutrophil and white blood-cell counts. In addition, we have used transcriptome analysis to test the hypothesis that similar biological processes are altered by the different thiopurine-dosing strategies.

## METHODS

### Chemicals and Enzymes

The deoxythioguanosine (dTG) standard was from Carbosynth (Compton, United Kingdom); deoxyadenosine (dA) was from Sigma-Aldrich and deuterated methylmercaptopurine (internal standard) from Toronto Research Chemicals (Ontario, Canada). HPLC-grade formic acid was from Fisher Scientific (Loughborough, United Kingdom). All other reagents were from Sigma (Gillingham, United Kingdom). Calf intestinal alkaline phosphatase and nuclease P1 from *Penicillium citrinum* were from Sigma.

### Study Population and Sample Collection

Clinical samples were provided from adult IBD patients treated with either AZA or LDAA at 8 centers in the UK (see Table 1, Supplemental Digital Content 1, http://links.lww.com/IBD/B503). Patients treated with LDAA were from East Surrey Hospital (ESH) where patients were normally treated with LDAA as a first-line therapy, and without preselection based on RBC metabolites.^12^ The numbers of adult patients (ESH patient numbers in parentheses) being treated for IBD comprized 81 (43) with Crohn's Disease, 57 (28) with ulcerative colitis, 4 (1) with indeterminate colitis, and 3 (3) with microscopic colitis. At all centers, patients treated with AZA were treated according to standard clinical guidelines.^13^ Of the 145 patients, 84 (50 AZA and 34 LDAA patients) were not prescribed other medication; the remaining 61 patients were receiving a variety of additional drugs, including mesalamine (5-aminosalicylic acid; 34 patients), infliximab (4 patients, 1 of whom was receiving mesalamine as well) and/or steroids (prednisolone; 3 in the LDAA group and 7 in the AZA group); other drugs included statins, antidepressants, and proton pump inhibitors. Data were analyzed only with respect to thiopurine treatment status, using patients treated with LDAA at ESH, and patients treated with AZA across all centers; to avoid bias, LDAA/AZA data from ESH were also analyzed separately. As part of routine clinical practice some participating centers requested RBC thiopurine metabolite measurements (RBC-TGN and RBC-MMP) at the same time as samples were taken for dTG^DNA^ analysis; most (92%) of the samples with these data were from ESH.

### Ethical Statement

The study protocol was approved by NRES Committee South West—Cornwall & Plymouth, Bristol Research Ethics Committee Centre, and each patient gave written informed consent.

### Sample Preparation and LC-MS/MS Analysis of Deoxythioguanosine Incorporated in DNA (dTG^DNA^)

DNA was isolated from whole blood collected in EDTA tubes, using previously published methods,^14^ and the ratio of dTG relative to dA in DNA (expressed as the number of moles of dTG/1 × 10^6^ moles of dA) was analyzed using a recently developed HPLC-mass spectrometry method.^15^ Briefly, chromatographic separation of dTG and dA was with a XSelect HSS T3 3.5 μm 4.6 × 100 mm (Waters) column with a VanGuard 3.5 μm 3.9 × 5-guard column maintained at 30°C. Analytes were eluted with mobile phases of 0.01 M aqueous 0.05% formic acid (A) and 0.05% formic acid in acetonitrile (B). The flow rate was 0.5 mL/minute, and the mobile phase system consisted of a starting condition of 1% buffer B increasing to 3% at 1.1 minutes, 8% at 2.4 minutes, and increasing to a maximum of 30% at 4.1 minutes then decreasing to 5% at 4.5 minutes, maintained until 5.5 minutes then decreasing to 1% for an equilibration period of 2.5 minutes. Standards ranged from 100 μg/mL dA and 2000 ng/mL dTG and were serially diluted in water to 0.39 μg/mL dA and 3.9 ng/mL dTG, respectively. High (40,000 ng/mL dA:400 ng/mL dTG), medium (4000 ng/mL dA:40 ng/mL dTG) and low (400 ng/mL dA:4 ng/mL dTG) matrix controls were prepared in triplicate. Standards, matrix controls, and samples were injected in a volume of 50 μL; for sample injections this is equivalent to 0.2 μg patient DNA per injection.

### RNA-seq Methods

Patients for RNA-seq analysis were being treated with the appropriate drug, were in clinical remission at 12 weeks, and for whom a pretreatment blood sample was available; samples for analysis were selected “blind” from the pool of patient samples that met these criteria. At the time of selection, other clinical data for the patients were not available. RNA-seq of total RNA from whole blood, collected into Tempus RNA tubes, was performed by AROS Applied Biotechnology (Aarhus, Denmark). Sequencing libraries were prepared in 2 batches, LDAA patients and AZA patients, each batch consisting of 10 samples representing 1 pretreatment and 1 post-treatment sample (12 weeks after initiation of thiopurine therapy) from each patient. Input RNA was 400 ng from each sample, and libraries were prepared using the Illumina TruSeq Stranded mRNA LT Sample preparation kit after fragmentation using the alternative Illumina fragmentation protocol and RiboZero rRNA depletion. Libraries were quality controlled with respect to concentration and size profile. Size profiles (Agilent Bioanalyzer) from samples tested were 300 to 700 bp; library and library pool concentrations were determined using the KAPA qPCR kit in triplicate reactions with 3 independent 1 × 10^6^-fold dilutions of libraries. RNA-seq libraries were sequenced on a HiSeq 2500 using Illumina SBS v4 chemistry using a paired-end 100-bp protocol. Base calls were converted and demultiplexed according to the sequencing primer barcodes, using Illumina CASAVA software, into FASTQ files. Paired-end reads (100 bp) in FASTQ files were aligned to UCSC hg19 using Bowtie 2.2^16^ and Tophat 2.0.13^17^ running under Linux (Ubuntu). Alignments were counted using Bioconductor R packages “GenomicRanges” and “GenomicAlignments.”

### Statistical Analyses

All statistical analyses were conducted using R 3.1.2 and 3.3.0.^18^ We regard these analyses as exploratory and have not applied corrections for experiment-wise error rate in rank correlation and rank sum tests applied to different variable and data subsets. Nonparametric methods (2-sided; Kendall's Rank Correlation, Asymptotic Wilcoxon Mann–Whitney Rank Sum Test, Kruskal-Wallis Test) were used for basic analyses of thiopurine metabolites and clinical correlates. Parametric methods (1- and 2-sample [Welch's] *t* tests) were used where data were normally distributed (Shapiro-Wilks test). For analysis of dTG^DNA^ at different times after treatment initiation, patients were divided into 2 groups: treatment time for 4 to 167 days and >167 days; the boundary at 167 days (24 wk) represents times to achieve remission (≤167 d) and maintenance of remission (>167 d) used in a recent clinical study.^19^

Gene-count data from RNA-seq studies were analyzed using a mixed-effect approach in “edgeR” to derive treatment effects (post/pre) across all patients in each group (standard-dose azathioprine, AZA, and low-dose azathioprine with allopurinol, LDAA).^20^ Genes >1.5-fold over- or underexpressed in response to drug treatment (Benjamini-Hochberg False Discovery Rate [BH FDR] < 0.05) were mapped separately to Gene Ontology (GO) biological processes using “goseq” in R. For the *goseq* analysis, a BH FDR of <0.001 was applied to the LDAA data and <0.05 to the AZA data. The lists of statistically significant GO terms returned by *goseq* were summarized graphically using the web resource Revigo,^21^ using a similarity cutoff of 0.7, and Cytoscape.^22^ Robustness of the *goseq* analyses was tested by applying a jack-knife procedure, running the *goseq* analysis on the *edgeR* output after excluding each patient in turn. Summary lists of genes differentially expressed in common with both treatments were mapped to GO terms using “BiomaRt” in R and the GO terms summarized using Revigo using a similarity score of 0.4.

## RESULTS

### Incorporation of dTG into DNA of Nucleated Blood Cells

**FIGURE 2.**
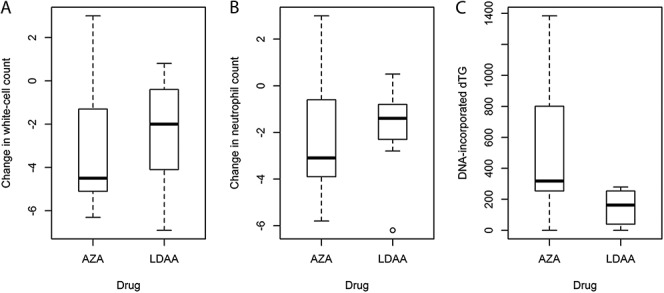
Changes in white blood-cell counts (A), neutrophil counts (B), and levels of dTG^DNA^ (C) in newly diagnosed patients at 12 weeks after therapy with AZA or LDAA. The data show a significant reduction in mean white-cell and neutrophil counts (neutrophils in brackets) from 9.3 (6.4) to 6.6 (4.4) as a result of therapy with AZA or LDAA (n = 18; one-sample *t*-test on difference per patient, *P* ≤ 0.001), but no significant difference between the AZA or LDAA treatments (*t*-test; *P* ≥ 0.64). However, there were significantly lower dTG^DNA^ levels in the LDAA group (Mann–Whitney Rank Sum Test, Z = 2.21, *P* = 0.03). Units: neutrophil and white blood-cell counts × 10^9^/L; dTG^DNA^, moles dTG/10^6^ moles dA.

Eighteen samples with data on white blood-cell counts were received from newly diagnosed patients with IBD at initiation of treatment with AZA or LDAA (9 patients per group); these patients were not prescribed additional medication. Twelve weeks after initiation of therapy, there was a significant reduction in neutrophil and white-blood cell counts (Fig. 2; 1-sample *t* test, *P* ≤ 0.003, n = 18); 3 patients in the AZA group were leukopenic (white-blood cell counts < 4 × 10^9^/L). There was no significant difference in reduction of neutrophil or white-blood cell count between the 2 drug regimens (Welch's *t* test; *P* ≥ 0.64), although dTG^DNA^ for patients in the LDAA group was significantly lower (Mann–Whitney test, *P* = 0.03; Fig. 2).

**FIGURE 3.**
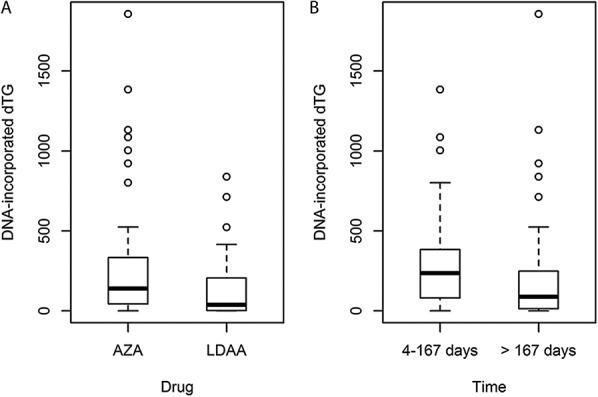
A, Box and whisker plot of data for dTG^DNA^ in the AZA and LDAA treatment groups for all patients in the study and sampled at different periods on therapy. For AZA (all sites) median dTG^DNA^ was 139.6 (mean 241.5, range 0–1857, SD 310.7, n = 97) and for LDAA-treated patients (ESH) 37.6 (mean 128.8, range 0–839.3, SD 184.5, n = 48). For comparison, AZA-treated patients at ESH alone had median dTG^DNA^ 173.1 (mean 308.7, range 0–1384.5, SD 399.4, n = 27). B, Box and whisker plot for dTG^DNA^ in 2 time periods: 4 to 167 days (∼≤ 24 wk) and > 167 days (>24 wk), representing time to achievement of remission, and maintenance of remission.^19^ Initial assessment of dTG^DNA^ by time periods at 4 to 83, 84 to 167, 168 to 252, or >252 days after initiation of therapy suggested a decrease after 167 days (see Fig. 1, Supplemental Digital Content 1, http://links.lww.com/IBD/B503); therefore, periods 1 and 2, and 3 and 4 were combined for analysis. The distributions of treatment times were similar for AZA and LDAA-treated patients. Units: dTG^DNA^, moles dTG/10^6^ moles deoxyadenosine.

For the dataset as a whole, dTG^DNA^ was also lower in the patients receiving LDAA compared with those on AZA (Mann–Whitney test, Z = 2.95, *P* = 0.003; Fig. 3), but there was no significant difference in white-blood cell counts between the 2 treatments (Mann–Whitney, *P* = 0.65). With respect to time of treatment, dTG^DNA^ levels in patients receiving either AZA or LDAA for more than 24 weeks (>167 d; maintenance of remission) were lower than for patients who had received therapy for up to 24 weeks (4–167 d; achievement of clinical remission) (*P* ≤ 0.01 for all data and ESH data alone; Fig. 3). There were no patients deficient in TPMT activity (<10 mU/L), and no relationship between dTG^DNA^ and patient TPMT activity, either for the whole dataset or each drug treatment group alone (Kendall, *P* > 0.5).

Patients on LDAA received azathioprine at approximately one-quarter the dose of those on AZA (median 40.5 mg [range 15–75], and 125 mg [range 22–250] per day, averaged across a week, for LDAA and AZA, respectively). For patients receiving AZA, there was a significant correlation between dose and dTG^DNA^ (*P* = 0.012, Table 1), but this was marginal for LDAA patients (*P* = 0.073). In some patients within both AZA and LDAA treatment groups, dTG^DNA^ was below the level of detection; for the 12 patients with undetectable dTG^DNA^ where RBC TGN measurements were available, RBC TGN levels ranged between 194 and 1016 pmol/8 × 10^8^ RBC, indicating that undetectable dTG^DNA^ was not a result of poor compliance. Furthermore, there were no differences in white-blood cell counts between patients with detectable dTG^DNA^ and those without (Mann–Whitney, *P* = 0.96, AZA and LDAA groups combined).

**TABLE 1.**
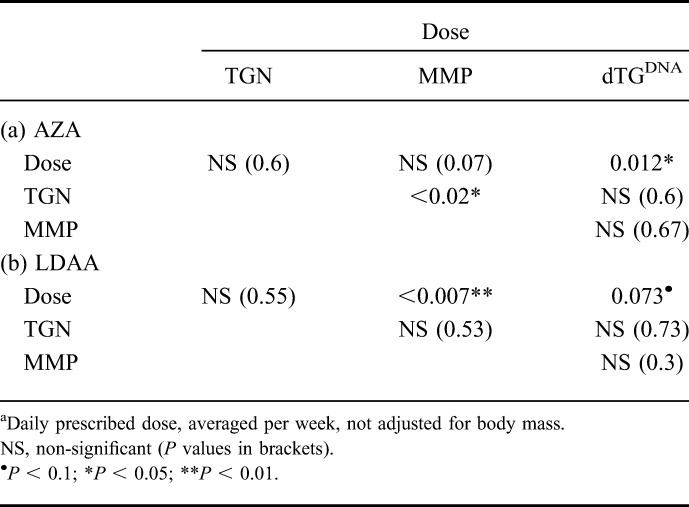
Probabilities for Kendall Rank Correlations Between Azathioprine Dose^a^, RBC TGN, RBC MMP and dTG^DNA^ for the AZA (a) and LDAA (b) Drug Treatment Groups (n = 97 and 48, Respectively)

### RBC Metabolites and Relationships with dTG^DNA^

With respect to the relationships between RBC-TGNs and -MMPs, there was no correlation between RBC-TGN and RBC-MMP for LDAA patients, but the converse was true for AZA. However, there was a significant positive correlation between prescribed azathioprine dose and RBC-MMP levels for LDAA but not for AZA. Conversely, RBC-TGN showed no correlation with thiopurine dose for either AZA or LDAA. Finally, no significant relationship was apparent between dTG^DNA^ and RBC-TGNs or RBC-MMPs for patients on AZA or LDAA (Table 1).

RBC-TGN levels differed between patients in the 2 drug groups with patients on LDAA having higher median RBC-TGN levels (Mann–Whitney test, *P* = 0.008), although the effect was not significant for the ESH data alone (*P* = 0.062; Table 2). Although median and mean RBC-MMP levels were lower in the LDAA group, this difference was not statistically significant (*P* ≥ 0.26). In contrast to dTG^DNA^ measurements, there was no difference between the early and late time periods of treatment for either RBC metabolite (Mann–Whitney Rank Sum Test, *P* ≥ 0.35).

**TABLE 2.**
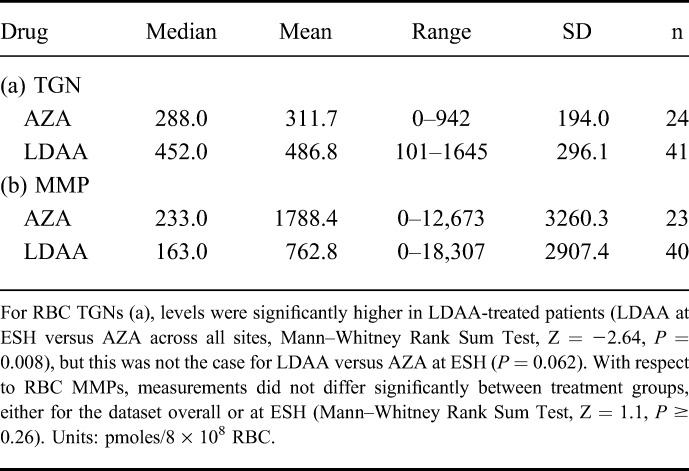
RBC-TGNs and MMP Measurements by Treatment Group

### Transcriptome Changes as Markers of Biological Response to Thiopurine Therapy

**FIGURE 4.**
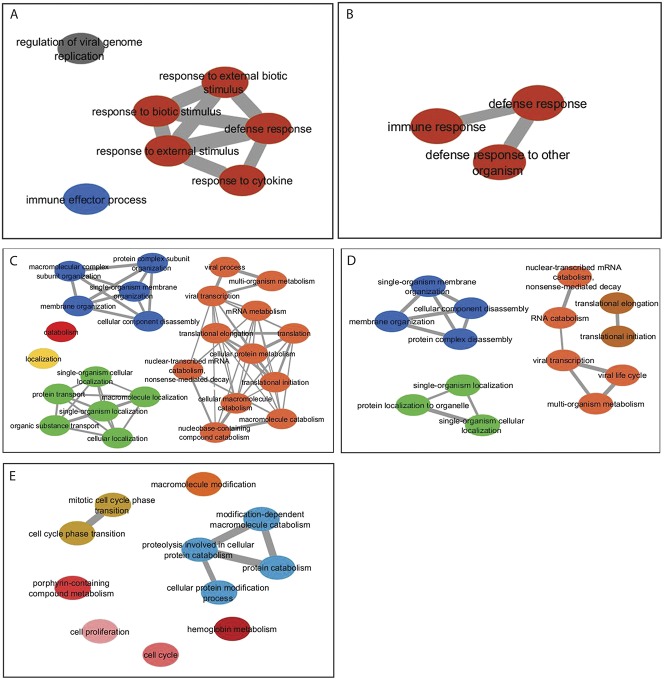
GO profiling from RNA-seq data for thiopurine-treated patients: (A) biological processes significantly associated with genes underexpressed as a consequence of LDAA therapy; (B) sampled (jack-knife: each patient excluded in turn) data, biological processes significantly associated with underexpressed genes in LDAA-treated patients which were common to all subsampled sets; (C) biological processes significantly associated with over-expressed genes in LDAA-treated patients; (D) biological processes significantly associated with overexpressed genes which were common to all subsampled sets of LDAA-treated patients; in A to D, the BH FDR was set to *P* < 0.001 for GO categories. E, GO profiling from RNA-seq data for AZA-treated patients, showing biological processes significantly associated with underexpressed genes (BH FDR was set to *P* < 0.05 for GO categories). No biological processes were significantly associated with increases in gene expression in response to AZA, but only the GO cell compartments “cell periphery” and “plasma membrane,” were significantly associated with increases in gene expression in response to AZA and these were common to all subsamples.

For 10 newly diagnosed patients, blood samples for transcriptome analysis by RNA-seq were collected prior to thiopurine therapy, and a second sample collected from each patient after 12 weeks of treatment with either AZA alone or LDAA; for these patients, 5 treated with AZA and 5 with LDAA, no adverse drug reactions were recorded, and all were in clinical remission. The time of sampling was consistent with achievement of maximal levels of dTG^DNA^ and 9 of the 10 patients had detectable dTG^DNA^; mean and median dTG^DNA^ levels were similar in the 2 groups. The patient without detectable dTG^DNA^ was receiving LDAA and RBC-TGN levels indicated compliance (Table 3). All LDAA patients were from 1 site (ESH) and were not on additional medication, whereas, the AZA patients were from 3 separate sites and some were receiving additional drugs (Table 3). For the LDAA patient group overall, 619 genes were repressed and 614 had higher expression in response to therapy (seeTables S2 and S3, Supplemental Digital Content 1, http://links.lww.com/IBD/B503); applying a BH FDR of <0.001 for Gene Ontology (GO) analysis of gene lists, underexpressed genes were significantly associated with biological processes of immune defense (Fig. 4; see Table S4, Supplemental Digital Content 1, http://links.lww.com/IBD/B503). Subsampling by reanalysis after exclusion of each patient in turn revealed a core set of biological-process GO categories comprising “*defence response*” (GO:0006952), “*defence response to other organism*” (GO:0098542) and “*immune response*” (GO:0006955). GO biological-process categories significantly associated with overexpressed genes in the LDAA-treated patients were more diverse, with subsampling giving RNA metabolic processes and processes of macromolecular localization and organization as the GO category core sets for biological processes (Fig. 4; see Table S5, Supplemental Digital Content 1, http://links.lww.com/IBD/B503). Within the top 5 genes repressed by LDAA (lowest *P* values), *SNRK-AS1* is potentially involved in colon-cell proliferation, *PLTP* modulates inflammation and immune responses and *C1orf56* (*MENT*) is a tumor modifier associated with lymphoma; the top 5 overexpressed genes were associated with activation of host-defense responses and hematopoiesis (see Tables S2 and S3, Supplemental Digital Content 1, http://links.lww.com/IBD/B503).

**TABLE 3.**
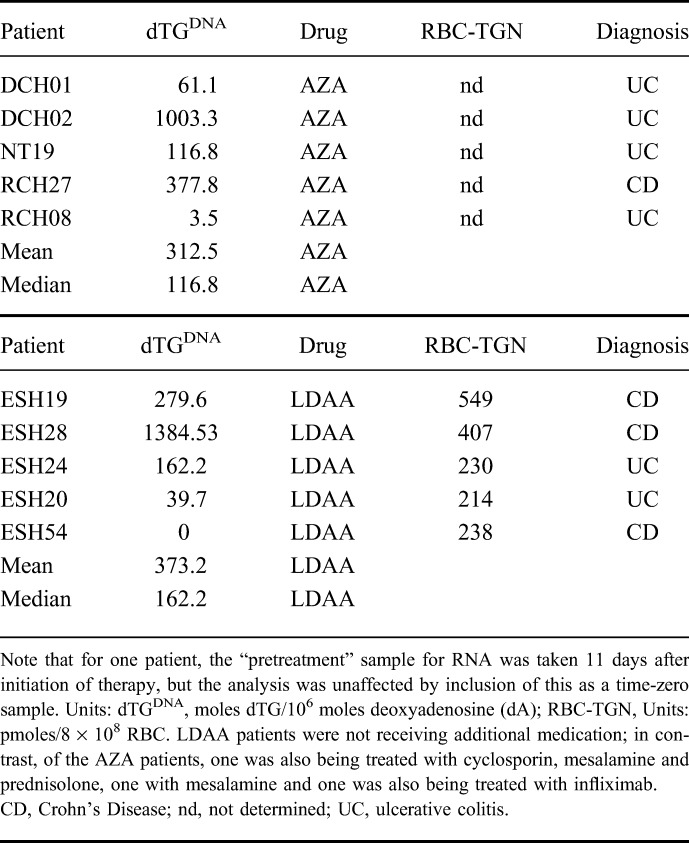
RNA-seq Samples

For the AZA-treated group, 464 genes were repressed and 165 had higher expression in response to therapy (see Tables S6 and S7, Supplemental Digital Content 1, http://links.lww.com/IBD/B503). In these gene lists, there was no significant representation in any GO categories at a BH FDR of <0.001. At an FDR of <0.05, underexpressed genes were enriched in diverse GO categories, particularly those concerned with ion transport and homeostasis, proteolysis and protein catabolism, and cell-cycle regulation (Fig. 4; see Table S8, Supplemental Digital Content 1, http://links.lww.com/IBD/B503). The RNA-seq data for the AZA patient group as a whole were less consistent than the LDAA group; 3 of the 5 jack-knife subsamples for underexpressed genes had no significant GO categories. Overexpressed genes in the complete AZA dataset fell into only 2 GO categories: plasma membrane and cell periphery cell compartments, with no representation in biological-process GO categories; these GO cell-compartment categories were also common to all subsamples. However, overexpressed genes in 3 of the 5 subsamples were involved in regulation of cell activation and immune system biological processes (see Table S9, Supplemental Digital Content 1, http://links.lww.com/IBD/B503).

The *RAC1* GTPase is thought to be a target of thiopurines^23^ but was not represented in the lists of genes differentially expressed in response to therapy (see Tables S2, S3, S6, and S7, Supplemental Digital Content 1, http://links.lww.com/IBD/B503). Furthermore, GO terms associated with *RAC1* function (http://www.genecards.org/cgi-bin/carddisp.pl?gene=RAC1) were not included in the lists of GO terms significantly associated with differentially expressed genes.

Thirty repressed and 27 overexpressed genes were common to both treatments (see Table S10, Supplemental Digital Content 1, http://links.lww.com/IBD/B503). Amongst the common underexpressed genes we highlight guanylate cyclase 2C (*GUCY2C*), a gene predominantly expressed in colon epithelial cells^24^ which was repressed by up to 2.5-fold (LDAA: *P* < 0.0001; AZA: *P* = 0.03). After mapping to GO terms and summarizing the GO term lists, common over- and underexpressed genes were associated mainly with oxidation-reduction processes (GO:0055114; frequency >15% of genes). In addition, underexpressed genes were also associated with DNA metabolic (GO:0006259; 6.3%) and phosphorylation (GO:0016310; 6.3%) processes, and overexpressed genes (see Table S11, Supplemental Digital Content, http://links.lww.com/IBD/B503) with processes of transport (GO:0006810; 17.4%), carbohydrate metabolism (GO:0005975; 6%), stress response (GO:0006950; 4.1%), and nucleic acid phosphodiester bond hydrolysis (GO:0090305; 2.5%).

## DISCUSSION

These data confirm the measurable incorporation of thioguanine nucleotides into the DNA of nucleated blood cells of patients with IBD on thiopurine therapy. A clinical effect of thiopurines is the reduction of neutrophil and white cell counts^25–27^ which was clearly demonstrated with no difference between AZA and LDAA treatment groups; in contrast, dTG^DNA^ levels were lower in LDAA-treated patients, as shown by the treatment group sampled after 12 weeks of therapy and the full study dataset. Given correlations between thiopurine dose and dTG^DNA^ within treatments, lower dTG^DNA^ levels in the LDAA group may reflect the lower thiopurine dose. These data suggest that the biological effects of AZA or LDAA are independent of dTG^DNA^ levels in nucleated blood cells, in agreement with other studies showing no correlation between dTG^DNA^ and white-blood cell counts.^28^ Maximum dTG^DNA^ levels were in patients sampled within 24 weeks of the start of therapy, with lower levels in patients sampled at longer times after therapy initiation; lower levels of dTG^DNA^ on maintenance of remission may result from adaptation to thiopurines with respect to pathways of metabolism and rates of cell renewal in relevant target tissues.

In contrast to a previous study,^28^ we found no significant correlation between dTG^DNA^ and RBC-TGNs or RBC-MMPs. Furthermore, RBC-TGN levels were higher in LDAA-treated patients, in contrast to the lower dTG^DNA^ levels in this patient group. Our current understanding is that inhibition of xanthine oxidase by allopurinol reduces the metabolic inactivation of thiopurines, allowing more conversion to TGNs.^29^ MMPs, methylation products of TPMT activity, decrease in the presence of allopurinol by an unknown mechanism; TPMT inhibition by an allopurinol metabolite^30^ or the 6-MP metabolite thioxanthine,^29,31,32^ have been suggested, and depletion of phosphoribosylpyrophosphate or competitive inhibition of membrane transport processes are possible alternative mechanisms.^33^ Clearly, in our study the RBC-TGN and RBC-MMP levels varied between the AZA and LDAA treatment groups as expected.

It may be paradoxical that dTG^DNA^ levels in nucleated blood cells of LDAA-treated patients were low despite higher levels of RBC TGNs compared to patients treated with AZA. Nucleoside triphosphates are toxic to cells,^34^ and homeostatic mechanisms may counter raised TGTP levels by conversion of nucleoside triphosphates to monophosphates, as we have found with TGTP in vitro (Coulthard, McGarrity, Redfern; unpublished data), thus reducing the incorporation of dTG into DNA despite higher TGN levels. However, any interpretation of how dTG^DNA^ relates to RBC TGNs has to be speculative given our limited understanding of thiopurine metabolism in white blood cells and transfer of thiopurine metabolites between cell and tissue compartments. Indeed, the lack of a relationship between dTG^DNA^ and RBC-TGN has echoes of studies showing a lack of correlation between RBC-TGN levels and biological or clinical responses.^35–37^ RBCs have low IMPDH activity^38,39^ and RBC-TGNs must, therefore, be derived from liver thioguanine metabolized from azathioprine, or remain from immature erythrocytes. Further studies are needed to test the relationships between dTG^DNA^ and biological and clinical responses in IBD, and how dTG^DNA^ levels are affected by the dynamics of thiopurine and purine metabolism in different cell populations.

Although thiopurines are used extensively as immunosuppressants there is uncertainty of the mechanisms involved, a question that can be addressed by transcriptome analysis of peripheral blood with respect to immune system markers. Inhibition of GTP signaling, specifically the *RAC1* GTPase which is elevated in expression in activated T-cells,^40^ may be one mechanism, at least in vitro.^23,41,42^ However, evidence that this underlies clinical responses is lacking^43^ and interference in *RAC1* signalling was not apparent from our RNA-seq data. We found a clear response with respect to the downregulation of genes involved in host-defense mechanisms against microorganisms in LDAA patients. This may result from gene downregulation in relevant immune cells, or from a thiopurine-induced reduction of specific immune-cell populations. Whatever the mechanism, this is consistent with a role of microorganisms and host-defense interactions in the etiology of IBD. The wide range of dTG^DNA^ values despite a fairly homogenous LDAA dataset with respect to GO categories affected implies that dTG^DNA^ may not be a good biological marker of response.

Despite the similar range of dTG^DNA^ levels to the LDAA samples, the AZA-treated patients did not show a consistent transcriptome-response profile. While more extensive studies are needed to test for potentially confounding effects of bias from the treatment site, sample batch with respect to the LDAA/AZA comparisons or the effects of additional drugs on some patients in the AZA group, the Gene Ontology analysis, both for the shared and the full lists of differentially expressed genes, and the small number of shared genes up- or downregulated by either treatment, support the view that there are differences between LDAA and AZA in their underlying therapeutic mechanisms. An important caveat for the interpretation of the LDAA data is that allopurinol may have anti-inflammatory properties, independently of its effects on thiopurine metabolism, by scavenging free radicals, or reducing the T-cell activation^44^ involved in the pathogenesis of IBD.^45^ Thus, by providing complementary anti-inflammatory effects, the therapeutic value of allopurinol for IBD may extend beyond merely improving thiopurine pharmacokinetics.

Two differentially expressed genes may warrant further investigation. Firstly, the downregulation of *GUCY2C* was common to both treatments. This gene is well known for its role in endotoxin-mediated diarrhoea,^24^ is a potential colorectal cancer marker,^46^ and may be a link between obesity and colorectal cancer.^47^ Although *GUCY2C* expression has been detected in CD34^+^ progenitor cells in peripheral blood,^48^ a possible reflection of immune system status, the presence of *GUCY2C* transcripts in peripheral blood may also be a consequence of IBD disease processes. The detection and significant downregulation of *GUCY2C* transcripts in the LDAA and AZA patient groups raises questions about the potential of *GUCY2C* as a marker of IBD disease status and response to therapy with thiopurines or other drugs. Secondly, *MENT* is associated with lymphomagenesis in mice, and decreased methylation activity upregulates *MENT* expression.^49^ Noting that thiopurines decrease DNMT activity,^50^ and aside from the possibility that lower dTG^DNA^ with LDAA may reduce secondary cancer risk, the evidence for downregulation of *MENT* by LDAA suggests that the elevated lymphoma risk associated with thiopurine therapy in elderly patients^51^ may be avoided using LDAA rather than standard-dose azathioprine.

Whatever the mechanism of LDAA versus AZA, recent data from a small randomized clinical trial suggest that patients treated with an allopurinol and azathioprine combination have better response rates than those on AZA.^52^ Lower dTG^DNA^ and MMP levels in LDAA-treated patients suggest that LDAA as a first-line therapy may be the most appropriate for IBD patients, obviating the need for preselection on the basis of RBC metabolites.^12^ Further studies of the potential beneficial role of LDAA in different clinical contexts, such as a reduction in cancer risk, and to identify the precise mechanisms of action, are badly needed, but there is increasing evidence that this could be the initial treatment of choice for patients with IBD for whom immunosuppression therapy is appropriate.

## References

[1] Ansari A Elliott T Baburajan B Long-term outcome of using allopurinol co-therapy as a strategy for overcoming thiopurine hepatotoxicity in treating inflammatory bowel disease. Aliment Pharmacol Ther. 2008;28:734–741. 19145729 10.1111/j.1365-2036.2008.03782.x

[2] Ansari A Patel N Sanderson J Low-dose azathioprine or mercaptopurine in combination with allopurinol can bypass many adverse drug reactions in patients with inflammatory bowel disease. Aliment Pharmacol Ther. 2010;31:640–647. 20015102 10.1111/j.1365-2036.2009.04221.x

[3] Sparrow MP Hande SA Friedman S Effect of allopurinol on clinical outcomes in inflammatory bowel disease nonresponders to azathioprine or 6-mercaptopurine. Clin Gastro Hepatol. 2007;5:209–214. 10.1016/j.cgh.2006.11.020 17296529

[4] Sparrow MP Hande SA Friedman S Allopurinol safely and effectively optimizes tioguanine metabolites in inflammatory bowel disease patients not responding to azathioprine and mercaptopurine. Aliment Pharmacol Ther. 2005;22:441–446. 16128682 10.1111/j.1365-2036.2005.02583.x

[5] Cuffari C Theoret Y Latour S 6-Mercaptopurine metabolism in Crohn's disease: correlation with efficacy and toxicity. Gut. 1996;39:401–406. 8949645 10.1136/gut.39.3.401 PMC1383347

[6] Dubinsky MC Lamothe S Yang HY Pharmacogenomics and metabolite measurement for 6-mercaptopurine therapy in inflammatory bowel disease. Gastroenterology. 2000;118:705–713. 10734022 10.1016/s0016-5085(00)70140-5

[7] Dubinsky MC Yang H Hassard PV 6-MP metabolite profiles provide a biochemical explanation for 6-MP resistance in patients with inflammatory bowel disease. Gastroenterology. 2002;122:904–915. 11910342 10.1053/gast.2002.32420

[8] Relling MV Hancock ML Rivera GK Mercaptopurine therapy intolerance and heterozygosity at the thiopurine S-methyltransferase gene locus. J Natl Cancer Inst. 1999;91:2001–2008. 10580024 10.1093/jnci/91.23.2001

[9] Coulthard SA Hogarth LA Little M The effect of thiopurine methyltransferase expression on sensitivity to thiopurine drugs. Mol Pharmacol. 2002;62:102–109. 12065760 10.1124/mol.62.1.102

[10] Hoentjen F Seinen ML Hanauer SB Safety and effectiveness of long-term allopurinol-thiopurine maintenance treatment in inflammatory bowel disease. Inflamm Bowel Dis. 2013;19:363–369. 22605661 10.1002/ibd.23021

[11] Smith MA Blaker P Marinaki AM Optimising outcome on thiopurines in inflammatory bowel disease by co-prescription of allopurinol. J Crohns Colitis. 2012;6:905–912. 22386736 10.1016/j.crohns.2012.02.007

[12] Pavlidis P Stamoulos P Abdulrehman A Long-term safety and efficacy of low-dose azathioprine and allopurinol cotherapy in inflammatory bowel disease: a large observational study. Inflamm Bowel Dis. 2016;22:1639–1646. 27271488 10.1097/MIB.0000000000000827

[13] Mowat C Cole A Windsor A Guidelines for the management of inflammatory bowel disease in adults. Gut. 2011;60:571–607. 21464096 10.1136/gut.2010.224154

[14] Daly AK Steen VM Fairbrother KS CYP2D6 multiallelism. Meth Enzymol. 1996;272:199–210. 8791778 10.1016/s0076-6879(96)72024-4

[15] Coulthard SA Berry P McGarrity S Liquid chromatography-mass spectrometry for measuring deoxythioguanosine in DNA from thiopurine-treated patients. J Chromatogr B Analyt Technol Biomed Life Sci. 2016;1028:175–180. 10.1016/j.jchromb.2016.06.017 PMC4955110 27362994

[16] Langmead B Trapnell C Pop M Ultrafast and memory-efficient alignment of short DNA sequences to the human genome. Genome Biol. 2009;10:R25. 19261174 10.1186/gb-2009-10-3-r25 PMC2690996

[17] Trapnell C Pachter L Salzberg SL TopHat: discovering splice junctions with RNA-Seq. Bioinformatics. 2009;25:1105–1111. 19289445 10.1093/bioinformatics/btp120 PMC2672628

[18] R: A Language and Environment for Statistical Computing [program]. Vienna, Austria: R Foundation for Statistical Computing; 2014.

[19] Colombel JF Sandborn WJ Reinisch W Infliximab, azathioprine, or combination therapy for Crohn's disease. N Eng J Med. 2010;362:1383. 10.1056/NEJMoa0904492 20393175

[20] Anders S McCarthy DJ Chen Y Count-based differential expression analysis of RNA sequencing data using R and bioconductor. Nat Protoc. 2013;8:1765–1786. 23975260 10.1038/nprot.2013.099

[21] Supek F Bosnjak M Skunca N REVIGO summarizes and visualizes long lists of gene ontology terms. PLoS One. 2011;6:e21800. 21789182 10.1371/journal.pone.0021800 PMC3138752

[22] Shannon P Markiel A Ozier O Cytoscape: a software environment for integrated models of biomolecular interaction networks. Genome Res. 2003;13:2498–2504. 14597658 10.1101/gr.1239303 PMC403769

[23] Tiede I Fritz G Strand S CD28-dependent Rac1 activation is the molecular target of azathioprine in primary human CD4+ T lymphocytes. J Clin Invest. 2003;111:1133–1145. 12697733 10.1172/JCI16432 PMC152932

[24] Brierley SM Guanylate cyclase-C receptor activation: unexpected biology. Curr Opin Pharmacol. 2012;12:632–640. 23131468 10.1016/j.coph.2012.10.005

[25] Evans WE Horner M Chu YQ Altered mercaptopurine metabolism, toxic effects, and dosage requirement in a thiopurine methyltransferase-deficient child with acute lymphocytic leukemia. J Pediatr. 1991;119:985–989. 1960624 10.1016/s0022-3476(05)83063-x

[26] Park MS Kim DH Kim DH Leukopenia predicts remission in patients with inflammatory bowel disease and Behcet's disease on thiopurine maintenance. Dig Dis Sci. 2015;60:195–204. 25239495 10.1007/s10620-014-3355-4

[27] Wong DR Coenen MJ Vermeulen SH Early assessment of thiopurine metabolites identifies patients at risk of thiopurine-induced leukopenia in inflammatory bowel disease. J Crohns Colitis 2016;11:175–184. 27402913 10.1093/ecco-jcc/jjw130

[28] Cuffari C Li DY Mahoney J Peripheral blood mononuclear cell DNA 6-thioguanine metabolite levels correlate with decreased interferon-gamma production in patients with Crohn's disease on AZA therapy. Dig Dis Sci. 2004;49:133–137. 14992447 10.1023/b:ddas.0000011614.88494.ee

[29] Seinen ML van Asseldonk DP de Boer NKH The effect of allopurinol and low-dose thiopurine combination therapy on the activity of three pivotal thiopurine metabolizing enzymes: results from a prospective pharmacological study. J Crohns Colitis. 2013;7:812–819. 23317929 10.1016/j.crohns.2012.12.006

[30] Duley JA Chocair PR Florin TH Observations on the use of allopurinol in combination with azathioprine or mercaptopurine. Aliment Pharmacol Ther. 2005;22:1161–1162. 16305731 10.1111/j.1365-2036.2005.02703.x

[31] Blaker PA Arenas-Hernandez M Smith MA Mechanism of allopurinol induced TPMT inhibition. Biochem Pharmacol. 2013;86:539–547. 23770457 10.1016/j.bcp.2013.06.002

[32] Curkovic I Rentsch KM Frei P Low allopurinol doses are sufficient to optimize azathioprine therapy in inflammatory bowel disease patients with inadequate thiopurine metabolite concentrations. Eur J Clin Pharmacol. 2013;69:1521–1531. 23588559 10.1007/s00228-013-1500-1

[33] Sparrow MP Use of allopurinol to optimize thiopurine immunomodulator efficacy in inflammatory bowel disease. Gastroenterol Hepatol. 2008;4:505–511. PMC3096137 21960930

[34] Batiuk TD Schnizlein-Bick C Plotkin Z Guanine nucleosides and Jurkat cell death: roles of ATP depletion and accumulation of deoxyribonucleotides. Am J Physiol Cell Physiol. 2001;281:C1776–C1784. 11698235 10.1152/ajpcell.2001.281.6.C1776

[35] Konidari A Anagnostopoulos A Bonnett LJ Thiopurine monitoring in children with inflammatory bowel disease: a systematic review. Br J Clin Pharmacol. 2014;78:467–476. 24592889 10.1111/bcp.12365 PMC4243898

[36] Lee MN Kang B Choi SY Relationship between azathioprine dosage, 6-thioguanine nucleotide levels, and therapeutic response in pediatric patients with IBD treated with azathioprine. Inflamm Bowel Dis. 2015;21:1054–1062. 25851563 10.1097/MIB.0000000000000347

[37] Osterman MT Kundu R Lichtenstein GR Association of 6-thioguanine nucleotide levels and inflammatory bowel disease activity: a meta-analysis. Gastroenterology. 2006;130:1047–1053. 16618398 10.1053/j.gastro.2006.01.046

[38] Guicherit OM Cooper BF Rudolph FB The muscle and nonmuscle isozymes of adenylosuccinate synthetase are encoded by separate genes with differential patterns of expression. Adv Exp Med Biol. 1994;370:585–590. 7660974 10.1007/978-1-4615-2584-4_122

[39] Montero C Duley JA Fairbanks LD Demonstration of induction of erythrocyte inosine monophosphate dehydrogenase activity in Ribavirin-treated patients using a high performance liquid chromatography linked method. Clin Chim Acta. 1995;238:169–178. 7586576 10.1016/0009-8981(95)06088-u

[40] Shin JY Wey M Umutesi HG Thiopurine prodrugs mediate immunosuppressive effects by interfering with Rac1 protein function. J Biol Chem. 2016;291:13699–13714. 27189938 10.1074/jbc.M115.694422 PMC4919453

[41] Marinkovic G Hamers AAJ de Vries CJM 6-Mercaptopurine reduces macrophage activation and gut epithelium proliferation through inhibition of GTPase Rac1. Inflamm Bowel Dis. 2014;20:1487–1495. 25029617 10.1097/MIB.0000000000000122

[42] Marinkovic G Kroon J Hoogenboezem M Inhibition of GTPase Rac1 in endothelium by 6-mercaptopurine results in immunosuppression in nonimmune cells: new target for an old drug. J Immunol. 2014;192:4370–4378. 24670805 10.4049/jimmunol.1302527

[43] Lev-Tzion R Renbaum P Beeri R Rac1 polymorphisms and thiopurine efficacy in children with inflammatory bowel disease. J Pediatr Gastroenterol Nutr. 2015;61:404–407. 25885881 10.1097/MPG.0000000000000820

[44] Perez-Mazliah D Albareda MC Alvarez MG Allopurinol reduces antigen-specific and polyclonal activation of human T cells. Front Immunol. 2012;3:295. 23049532 10.3389/fimmu.2012.00295 PMC3448060

[45] Monteleone G Caprioli F T-cell-directed therapies in inflammatory bowel diseases. Clin Sci. 2010;118:707–715. 20350293 10.1042/CS20100027

[46] Sargent DJ Shi Q Gill S Molecular testing for lymph node metastases as a determinant of colon cancer recurrence: results from a retrospective multicenter study. Clin Cancer Res. 2014;20:4361–4369. 24919572 10.1158/1078-0432.CCR-13-2659

[47] Lin JE Colon-Gonzalez F Blomain E Obesity-induced colorectal cancer is driven by caloric silencing of the Guanylin-GUCY2C paracrine signaling axis. Cancer Res. 2016;76:339–346. 26773096 10.1158/0008-5472.CAN-15-1467-T PMC4717834

[48] Fava TA Desnoyers R Schulz S Ectopic expression of guanylyl cyclase C in CD34+ progenitor cells in peripheral blood. J Clin Oncol. 2001;19:3951–3959. 11579116 10.1200/JCO.2001.19.19.3951

[49] Alkebsi L Handa H Sasaki Y DNMT3B7 expression related to MENT expression and its promoter methylation in human lymphomas. Leuk Res. 2013;37:1662–1667. 24094886 10.1016/j.leukres.2013.09.014

[50] Hogarth LA Redfern CPF Teodoridis JM The effect of thiopurine drugs on DNA methylation in relation to TPMT expression. Biochem Pharm. 2008;76:1024–1035. 18708030 10.1016/j.bcp.2008.07.026

[51] Taleban S Elquza E Gower-Rousseau C Cancer and inflammatory bowel disease in the elderly. Dig Liver Dis. 2016;48:1105–1111. 27289334 10.1016/j.dld.2016.05.006

[52] Kiszka-Kanowitz M Theede K Mertz-Nielsen A Randomized clinical trial: a pilot study comparing efficacy of low-dose azathioprine and allopurinol to azathioprine on clinical outcomes in inflammatory bowel disease. Scand J Gastroenterol. 2016;51:1470–1475. 27686002 10.1080/00365521.2016.1216589

